# Clinical Profile of Leg Symptoms and Reflux in the Legs From Varicose Veins Disease Reported at a Tertiary Healthcare Institute in Jharkhand, India

**DOI:** 10.7759/cureus.69138

**Published:** 2024-09-10

**Authors:** Ashwini Kumar, Nishit Ranjan, Shiv K Mudgal, Vipin Patidar

**Affiliations:** 1 General Surgery, All India Institute of Medical Sciences Deoghar, Deoghar, IND; 2 College of Nursing, All India Institute of Medical Sciences Deoghar, Deoghar, IND

**Keywords:** clinical grading, deep and superficial venous insufficiency, perforator incompetence, reflux in vein, varicose vein

## Abstract

Background

Varicose veins, affecting 8-10% of the population, are categorized as primary or secondary, depending on the etiology. The aim of the study is to assess the clinical profile of leg symptoms and reflux in the legs of varicose veins disease among patients reported in a tertiary hospital.

Patients and methods

An institution-based cross-sectional exploratory study comprising 56 patients with reflux in legs of varicose veins disease selected through purposive sampling, conducted in the OPD (outpatient department), general surgery, AIIMS (All India Institute of Medical Sciences) Deoghar. The standardized study instrument includes the Aberdeen varicose veins questionnaire, and patients’ symptoms and concerns questionnaire, followed by a brief clinical examination based on CEAP (clinical, etiological, anatomical, and pathophysiological) classification and VCSS (venous clinical severity score) for C5/6 and color-flow duplex imaging of both lower limbs. Descriptive and inferential statistics were used for data analysis using IBM SPSS Statistics for Windows, Version 23 (IBM Corp., Armonk, NY).

Results

Bilateral presentation of the disease had been seen in 57.1% (n=32) participants, and the common history of present illness reported by participants included swelling along the veins (n=55, 98.2%), pain (n=51, 91.1%), and discoloration (n=33, 58.9%). The clinical grading revealed that most participants, i.e., 87.5% (n=49) had C2 and 75% (n=42) had C3 disease. The majority of participants 96.4% (n=54) suffered from primary varicose veins. Furthermore, all subjects exhibited superficial vein involvement, along with one individual having deep vein involvement and a few participants (n=35, 62.5%) having perforators. With this, the majority of participants (n=55, 98.2%) showed reflux pathology.

Conclusion

Varicose veins typically afflict men in their 40s and 50s who work in positions of extended standing. Limited awareness in the Santhal Pargana division, Jharkhand state, India may result in poor healthcare outcomes. Improving public awareness may help lessen disease-related consequences.

## Introduction

Varicose veins, derived from the Latin word varicosus, are not only dilated but tortuous and dilated [1]. Physiologically it is characterized as reverse flow via a defective valve on Valsalva's maneuver [2]. Humans suffer from this ailment due to their proclivity for standing upright [3]. Varicose veins affect 8-10% of the general population which is classified as primary when they are caused by an inherent irregularity in the veinous wall and secondary when they are caused by deep and superficial venous insufficiency [4-6]. Prolonged standing, obesity, age, smoking, hard lifting, and pregnancy are all risk factors. Straining during bowel motions, a lack of fiber in the diet, inherited vein weakness, and aging, all increase the risks [7].

Varicose veins can cause an unattractive appearance, aching, heaviness, pruritus, and early fatigue in the afflicted leg. These symptoms are exacerbated by extended standing and sitting in a dependent position and are alleviated by elevating the leg above the heart level. A small degree of edema is frequently observed. Thrombophlebitis, hyperpigmentation, lipodermatosclerosis, ulceration, and bleeding from attenuated vein clusters are more severe symptoms.

Modern vein-saving techniques are evolving as a result of the vein's relevance in vascular restoration. Puncture, avulsion, excision, cautery, ligation, injection sclerotherapy, and stripping were all traditional therapies [8]. Elastic compression stockings ranging from 20 to 50 mmHg are essential for controlling symptoms of varicose veins. Interventions are required when symptoms persist despite compression treatment or in situations of lipodermatosclerosis/venous ulcers. Despite its frequency, the etiology is still unknown, and sensitive patient profiles are unknown. There is a pressing need for clear patient classification for specific therapies, as well as defined diagnostic/management frameworks [6-9].

Distinct geographical variations in varicose vein care, as found in India and developed nations such as the United Kingdom, further complicate issues [10]. According to German research, a considerable number of patients had reflux that does not involve the terminal valve, suggesting that traditional surgical therapy overtreats by removing an intact valve [11]. Sclerotherapy has been investigated even in areas where research is restricted, such as tribal areas of the state of Jharkhand, India.

The current study was intended to assess the risk factors and clinical profile of patients with varicosity of veins at a tertiary care center in Jharkhand. The goal was to integrate regional practices with worldwide criteria, giving healthcare practitioners a holistic picture.

## Materials and methods

Study design and participants

An institution-based cross-sectional exploratory study on an outpatient basis, general surgery department, was undertaken in March-November 2023 at tertiary healthcare facilities in AIIMS (All India Institute of Medical Sciences) Deoghar, Jharkhand. Based on the eligibility criteria the participants were screened and selected from the general surgery department after an explanation regarding the project and obtaining informed consent. During March 6, 2023, and November 28, 2023, a total of 71 patients with disease for research participation were contacted. There were 56 people who matched the criteria for eligibility and agreed to participate in the study. The study included patients of age more than 14 years with varicose veins. Patients with deep vein thrombosis and instances of varicose veins that had already been treated were excluded from the study.

Data collection procedure and instruments

Enrolled individuals were scheduled for an interview by the principal investigator during their visit to the general surgery department's OPD (outpatient department) area. During the interview, individuals were thoroughly questioned about their varicose vein profiles and history of current illness, as well as clinical grading and nature of disease using standardized tools. A standardized tool, the “Aberdeen varicose veins questionnaire” and the “patients’ symptoms and concerns questionnaire” was used to collect information on socio-demographic background and clinical history [12], followed by a brief clinical examination based on CEAP classification and VCSS for C5/6 and color-flow duplex imaging of both lower limbs. Clinical tests were done on patients with varicose vein issues, including the Brodie Tendelenberg test, multiple tourniquet test, Perthe's test, modified Perthe's test, Schwartz test, Pratt test, cough impulse test (Morissey's), Fegan's test, Homan's sign, and Mosses sign. Along with this, a thorough systemic examination is performed to identify any systemic pathology. An abdominal examination is performed to rule out any condition that might cause secondary varicose veins. A Doppler evaluation is performed while the patient is standing. Before beginning therapy, a physical examination and Doppler-assisted mapping of the venous system were performed. Some particular indications were seen, including saphenofemoral junction incompetence, sapheno-popliteal junction incompetence, perforator incompetence, a deep venous system, and the presence of aberrant or unidentified veins or perforators. The sites of incompetence were marked by indelible skin pencils.

Statistical analysis

All statistical analyses have been carried out using IBM SPSS Statistics for Windows, Version 23 (IBM Corp., Armonk, NY), by both descriptive and inferential statistics, on the basis of the study's objectives and hypothesis, and a master data sheet developed by the researcher to calculate the data. For categorical data, patient characteristics were provided as numbers and percentages, and for continuous variables as means with standard deviations (SD).

Ethical consideration

The Institutional Ethics Committee, AIIMS Deogarh approved this study with approval code 2022-52-EMP-02 (STS 2022-03024), dated July 22, 2022, which followed the ethical standards outlined in the Declaration of Helsinki (2013) and good clinical practice recommendations as well as selected under STS (short-term-studentship) through ICMR (Indian Council of Medical Research) with reference No. 2022-03024. The purpose and protocol of the study were presented to the participants. Consent ensures voluntary involvement, anonymity, and data confidentiality.

## Results

Table 1 revealed that the mean age of the respondents was 44 years, with the majority (n=19, 33.9%) lying between the ages of 36 and 46 years. The study population was primarily male (n=46, 82.1%) and long-term workers (n=37, 66.1%). A significant number of participants (n=25, 44.6%) belonged to the lower middle class, and the majority (n=18, 32.1%) had finished their schooling up to the tenth grade. Furthermore, the majority of participants (n=52, 92.9%) were married, with only a minor fraction (n=4, 7.1%) being single. A family history of varicose veins was found in 10.5% (n=6) of individuals. A portion of the participants were tobacco users (n=17, 30.4%), and alcohol users (n=6, 10.7%), while 57.1% (n=32) did not use any such substances and a small percentage of participants had a history of past surgical intervention (n=7, 12.5%).

**Table 1:** Sociodemographic characteristics of study participants in terms of frequency and percentage distribution (n=56). n; Number of participants.

Sociodemographic characteristics of participants	n	%
Age (years), mean 44 years		
14-24	4	7.1
25-35	10	17.9
36-46	19	33.9
47-57	12	21.4
More than 57	11	19.6
Gender		
Male	46	82.1
Female	10	17.9
Marital status		
Unmarried	4	7.1
Married	52	92.9
Occupation		
Long-standing worker	37	66.1
Intermediate standing workers	14	25.0
Minimal standing worker	5	8.9
Socio-economic status (Modified Kuppuswami Scale)		
Upper class	8	14.3
Upper middle class	11	19.6
Middle class	9	16.1
Lower middle class	25	44.6
Lower class	3	5.4
Education		
No formal education	8	14.3
Primary	1	1.8
Secondary	4	7.1
10th pass	18	32.1
12th pass	15	26.8
Graduation	8	14.3
Post-graduation	2	3.6
Family history		
Present	6	10.7
Absent	50	89.3
Substance use		
No	32	57.1
Khaini and tobacco product	17	30.4
Alcohol	6	10.7
Others	1	1.8
Medical history		
Present	22	39.3
Absent	34	60.7
Surgical history		
Present	7	12.5
Absent	49	87.5
Routine exercise		
Present	19	33.9
Absent	37	66.1

Table 2 displays in terms of clinical presentation, etiology, grading, types, and nature of varicose veins disease; 57.1% (n=32) participants had a bilateral presentation of the disease, and the common history of present illness reported by participants included swelling along the veins (n=55, 98.2%), pain (n=51, 91.1%), discoloration (n=33, 58.9%), night cramps (n=27, 48.2%), itching (n=25, 44.6%), and ulcer (n=25, 44.6%). The average age at the beginning of the disease was 38.8 years, and the mean duration of symptoms was 4.7 months.

**Table 2:** Clinical presentation, grading, and nature of disease among study participants in terms of frequency and percentage distribution (n=56). n; Number of participants, *; Multiple responses.

Sr No	Clinical presentation, grading, and nature of disease	n	%
1.	Clinical presentation^*^ (history of present illness)		
	Swelling along the veins	55	98.2
	Pain	51	91.1
	Itching	25	44.6
	Ulcer	25	44.6
	Night cramps	27	48.2
	Discoloration	33	58.9
2.	Etiology		
	Primary	54	96.4
	Secondary	2	3.6
3.	Clinical grading (clinical stage)^ *^		
	C1 (telangiectasia)	39	69.6
	C2 (varicose vein)	49	87.5
	C3 (edema)	42	75.0
	C4 (skin changes without ulceration)	31	55.4
	C5 (skin changes with healed ulcer)	16	28.6
	C6 (skin changes with ulcer)	26	46.4
4.	Parameters related to the nature of the disease		
4.1	Side		
	Unilateral	24	42.9
	Bilateral	32	57.1
4.2	System involvement		
	Superficial vein	56	100.0
	Deep Vein	1	1.8
	Perforator	35	62.5
4.3	Pathology		
	Reflux	55	98.2
	Obstruction	1	1.8
5.	Age of symptoms onset (mean)	38.8 year	
6.	Duration of symptoms (mean)	4.7 months	

The clinical grading of reflux disease in the legs revealed that the majority of patients had C2 (n=49, 87.5%) and C3 (n=42, 75%) disease. The majority of participants 96.4% (n=54) suffered from primary varicose veins. Furthermore, all subjects exhibited superficial vein involvement, along with one individual having deep vein involvement and a few participants (n=35, 62.5%) having perforators. With this majority of participants (n=55, 98.2%) showed reflux pathology.

Table 3 revealed an analysis of clinical presentation for both lower limbs among study participants, with the majority of participants having mild (occasional) pain in the left (n=21, 37.5%) and right lower limbs (n=22, 39.3%), 46.4% (n=26) having varicose in both the left and right lower limbs, 55.4% (n=31) and 69.6% (n=39) of participants having venous edema all time (morning) in the left and right lower limbs respectively. Most of them, 62.5% (n=35) and 57.1%, (n=32) had no skin pigmentation, inflammation, and induration in the left and right lower limbs, respectively, while 75.0% (n=42) and 69.6% (n=39) had no active ulcer in the left and right lower limbs, respectively, indicating that the majority were not using compression therapy.

**Table 3:** Analysis of clinical presentation of study participants in terms of frequency and percentage distribution (n=56). n; Number of participants.

Clinical presentation parameters	Left lower limb		Right lower limb	
	n	%	n	%
Pain				
No	18	32.1	15	26.8
Mild (occasional)	21	37.5	22	39.3
Moderate (daily)	16	28.6	14	25.0
Severe (daily r/w medicine)	1	1.8	5	8.9
Varicose				
No	13	23.2	10	17.9
Mild (few)	26	46.4	26	46.4
Moderate (multiple)	17	30.4	16	28.6
Severe (extensive)	0	0.0	4	7.1
Venous edema				
No	16	28.6	10	17.9
Mild (evening)	4	7.1	4	7.1
Moderate (afternoon)	5	8.9	3	5.4
All time (morning)	31	55.4	39	69.6
Skin pigmentation				
No	35	62.5	32	57.1
Mild or limited	1	1.8	1	1.8
Moderate (diffuse)	2	3.6	3	5.4
Severe (wide and old)	18	32.1	20	35.7
Inflammation				
No	35	62.5	32	57.1
Mild (cellulitis)	3	5.4	3	5.4
Moderate (cellulitis)	15	26.8	20	35.7
Severe (cellulitis)	3	5.4	1	1.8
Induration				
No	35	62.5	32	57.1
Mild (<5 cm)	7	12.5	3	5.4
Moderate (<1/3^rd^ gaiter zone)	7	12.5	14	25.0
Severe (>1/3^rd^ gaiter zone)	7	12.5	7	12.5
Active ulcer (number)				
No	42	75.0	39	69.6
Mild (1)	11	19.6	6	10.7
Moderate (>= 2)	3	5.4	11	19.6
Active ulcer size				
No	42	75.0	39	69.6
Mild (2 cm)	11	19.6	13	23.2
Moderate (2-6 cm)	3	5.4	4	7.1
Ulcer duration				
No	42	75.0	39	69.6
Mild (<3 months)	3	5.4	5	8.9
Moderate (3-12 months)	2	3.6	10	17.9
Severe (>12 months)	9	16.1	2	3.6
Compression therapy				
No	44	78.6	44	78.6
Mild (intermittent)	5	8.9	10	17.9
Moderate (most days)	6	10.7	1	1.8
Severe (fully comply)	1	1.8	1	1.8

## Discussion

Age distribution

In our study, we observed that the age group most frequently affected by varicose veins was 36-46 years old, predominantly representing individuals from the low-middle socioeconomic class who had completed their education up to the 10th grade. This finding contrasts with the studies conducted by Joseph et al. and Gupta et al., which reported common age groups of 40-50 years and 21-50 years, respectively, with a mean age of presentation at 46 years [13,14]. The variation in age distribution could potentially be attributed to patients' attitudes towards the disease, geographical factors, including altitude, and the severity of the illness at the time of hospital presentation. Furthermore, this age group is particularly noteworthy as it coincides with the onset of various comorbidities and encompasses the primary earning members of society.

Sex distribution

Our study revealed a higher proportion of men affected by varicose veins compared to women, consistent with findings in other studies [13,14]. Similar gender disparities were observed in studies conducted at Dr. D. Y. Patil Hospital, Pune [15], and Rohtak [16]. This skewed distribution may be attributed to the nature of activities commonly performed by individuals in the region, where men predominantly assume the role of sole wage earners, while women shoulder household responsibilities. Additionally, when compared to Western data, a lower number of Indian middle-class and lower-class women are affected by varicose veins, possibly due to reduced concerns about cosmetic appearance. Consultation typically occurs only when the condition severely impacts their daily activities.

Occupation

Our investigation found that individuals engaged in occupations requiring prolonged periods of standing (more than six hours) were the most commonly affected by varicose veins. This observation aligns with the findings of Joseph et al., where 59.4% of patients had jobs involving extended standing hours [13]. Prolonged standing and strenuous muscle activity inherent in such professions can lead to the reflux of blood down the legs, consequently increasing vein pressure. Furthermore, lower-middle-class individuals were the most affected socioeconomic group, consistent with a study by Natour et al. [17]. This association may be attributed to limited knowledge about the disease within these populations and their prolonged ignorance due to inadequate education, as our study indicated that most individuals had received only basic education, typically up to the matriculation level.

Limb involvement

Our study identified the superficial vein as the most commonly affected, with only one case involving deep venous thrombosis. Doppler studies indicated that reflux was the pathophysiological factor in 98.24% (n=55) cases. Bilateral lower limb presentation was predominant, followed by unilateral right and unilateral left presentations. These findings were similar to those reported by Nayak et al. [18] but differed from the findings of Joseph et al. [13], where 37.1% had bilateral presentations. In our tertiary institute-based study, patients might have presented at an advanced disease stage, explaining the prevalence of bilateral presentations.

Clinical presentation

The most frequent patient complaint was swelling along the veins followed by pain. Pain was more prevalent in our study than in the study by Joseph et al., which reported a lower percentage [13]. Our findings underscore that Indian populations primarily seek treatment for varicose veins due to pain and subsequent complications, rather than cosmetic concerns. Additionally, complaints such as discoloration as skin changes were observed in 58.9% (n=33) of cases, aligning closely with the findings of Joseph et al. (53.5%) and another study (32.5%) [13,18].

Active ulceration was noted in 44.6% of cases, which was lower than Joseph et al.'s findings (57.6%) [13]. The precise pathophysiology of ulcer development remains unestablished, but it is theorized that venous ulcers result from the trapping of white cells in capillaries affected by venous hypertension. Endothelial cell activation due to hypoxia causes leucocytes to enter surrounding tissue, causing further harm. This destructive effect is intensified by capillary flow obstruction due to adherent leucocytes and thrombus formation from activated platelets. Venous hypertension is the primary factor contributing to ulcer genesis, exacerbated by an upright posture. Ambulatory venous hypertension is currently the only accepted underlying cause of venous ulceration, explaining the absence of venous ulcers in the upper limbs. The higher number of participants presenting with ulcers may be attributed to ignorance about the disease in its early stages, leading to complications.

Superficial veins were involved in all participants, while perforators were implicated in 39.3% of cases and deep veins in 1.8%. In contrast, Joseph et al. reported involvement in 72.4%, 0.6%, and 36.5% of cases for superficial veins, deep veins, and perforators, respectively [13]. These disparities suggest that most patients with venous disease primarily experience issues related to vein wall structure, often confined to the superficial veins. The mechanism initiating changes in vein wall structure remains poorly understood, although it is characterized by valvular failure, allowing retrograde flow within the veins due to gravity (venous incompetence). Importantly, it is no longer believed that venous incompetence results from primary mechanical valvular failure.

Primary varicose veins were observed in 96.4% of cases, while secondary varicose veins were present in 3.6% of cases. In contrast, a study reported 22% and 76% for primary and secondary varicose veins, respectively [19]. Primary varicose veins develop due to inherent vein wall weakness, while secondary varicose veins may result from trauma or deep vein thrombosis. Congenital varicose veins, constituting a small percentage, stem from disorders in natural venous system development and are typically part of limb vascular malformations present at birth. Regardless of the cause, defective venous valves can lead to venous blood stagnation in the leg, increasing vein pressure and potentially resulting in further varicose vein enlargement, symptomatic complications, and skin changes, including ulcer formation. Blockage of pelvic veins can exacerbate varicose vein effects, necessitating distinct treatment approaches.

Family association

This study identified a family history of varicose veins in 10.7% of cases. In contrast, Gupta et al. reported a family history in only 3.5% of cases, while Nayak et al. reported a family history in 12.5% of patients [14,18]. In our study, a family history did not significantly impact disease development.

This study revealed that 30.4% of participants used tobacco, whereas Joseph et al. reported tobacco use in 20% of participants [13]. Additionally, 10.7% of participants used alcohol, compared to the report by Joseph et al. of alcohol use in 27.6% of cases [13]. Our data was insufficient to establish a significant impact of tobacco on disease development, although different studies suggested that smokers had a 1.8 times greater risk of varicose veins [20-24]. However, the exact role of tobacco in varicosity remains unclear.

Limitation

Since this was a hospital-based study on a small scale, the results cannot be applied to the general population.

## Conclusions

The study on varicose veins disease in the Santhal Pargana division of the state of Jharkhand, India, found that the majority of instances occur in middle-aged males who work in jobs that require extended standing. The lack of awareness and healthcare utilization in this area contributes to late presentations and severe illness stages. Patients often complain of swelling and discomfort, with basic varicose veins and reflux being the most frequent. Compression stockings and limb elevation are both effective conservative therapies. The study emphasizes the need to increase public information in reducing the severity of instances and their accompanying problems. Future initiatives should prioritize early detection and preventative methods to reduce the burden of varicose veins on afflicted persons.
